# Root and rhizosphere contribution to the net soil COS exchange

**DOI:** 10.1007/s11104-023-06438-0

**Published:** 2023-12-23

**Authors:** Florian Kitz, Herbert Wachter, Felix Spielmann, Albin Hammerle, Georg Wohlfahrt

**Affiliations:** Universität Innsbruck, Institut für Ökologie, Sternwartestraße 15, Innsbruck, 6020 Austria

**Keywords:** Carbonyl sulfide, Soil gas exchange, Beech tree roots

## Abstract

**Background and aims:**

Partitioning the measured net ecosystem carbon dioxide (CO_2_) exchange into gross primary productivity (GPP) and ecosystem respiration remains a challenge, which scientists try to tackle by using the properties of the trace gas carbonyl sulfide (COS). Its similar pathway into and within the leaf makes it a potential photosynthesis proxy. The application of COS as an effective proxy depends, among other things, on a robust inventory of potential COS sinks and sources within ecosystems. While the soil received some attention during the last couple of years, the role of plant roots is mostly unknown. In our study, we investigated the effects of live roots on the soil COS exchange.

**Methods:**

An experimental setup was devised to measure the soil and the belowground plant parts of young beech trees observed over the course of 9 months.

**Results:**

During the growing season, COS emissions were significantly lower when roots were present compared to chambers only containing soil, while prior to the growing season, with photosynthetically inactive trees, the presence of roots increased COS emissions. The difference in the COS flux between root-influenced and uninfluenced soil was fairly constant within each month, with diurnal variations in the COS flux driven primarily by soil temperature changes rather than the presence or absence of roots.

**Conclusion:**

While the mechanisms by which roots influence the COS exchange are largely unknown, their contribution to the overall ground surface COS exchange should not be neglected when quantifying the soil COS exchange.

**Supplementary Information:**

The online version contains supplementary material available at 10.1007/s11104-023-06438-0.

## Introduction

The trace gas carbonyl sulfide (COS) has kept the scientific community interested in its properties for decades. Earlier COS research concentrated on its contribution to stratospheric aerosol formation (Crutzen 1976), newer studies focus on its potential as a tracer for plant photosynthesis and stomatal conductance (Sandoval-Soto et al. 2005; Seibt et al. 2010; Whelan et al. 2018). Leaves take up COS in the same way they take up CO_2_, through their stomata, and both gases pass through the mesophyll to their respective endpoints, carbonic anhydrase (CA) for COS and Rubisco for CO_2_ (Seibt et al. 2010). The key difference between COS and CO_2_, and the advantage scientists want to leverage during recent years, is that there is no known diffusion of COS out of the leaf, resulting in an unidirectional flux (Seibt et al. 2010; Stimler et al. 2010) in parallel with gross photosynthesis. Attempts have been made to use the unidirectional nature of the COS flux to estimate the gross CO_2_ uptake (Asaf et al. 2013; Berkelhammer et al. 2014; Berry et al. 2013; Billesbach et al. 2014; Blonquist et al. 2011; Maseyk et al. 2014; Whelan et al. 2018), especially as the later cannot be directly measured. Fluxes with opposing signs result in the net ecosystem productivity, which can be observed, but is hard to decompose into its component fluxes. The problem caused by multiple sinks and sources contributing to the observable net CO_2_ exchange is particularly relevant on scales larger than the leaf level (Anav et al. 2015).

In order to use COS as a proxy for the CO_2_ exchange of leaves, a robust understanding of other COS sinks and sources is needed. At the ecosystem level, the soil has received increased attention in regard to its COS exchange potential during the last couple of years (Bunk et al. 2017; Kaisermann et al. 2018; Kitz et al. 2020; Meredith et al. 2018; Ogee et al. 2016; Sun et al. 2015, 2018; Whelan et al. 2016), considering it harbors a multitude of organisms that could potentially contribute to the net ecosystem COS flux, via emission or uptake, and thus obscure or exaggerate part of the plant-mediated COS uptake. Other potential contributors to the ecosystem-scale COS exchange include plant litter, living non-leaf plant parts, and abiotic COS sources and sinks.

Oxic soils are usually thought to act as sinks for COS, which has been shown in the laboratory (Kesselmeier et al. 1999; Meredith et al. 2019; Van Diest and Kesselmeier 2008; Whelan et al. 2016) and in situ experiments (Steinbacher et al. 2004; Yi et al. 2007). The suspected mechanism underlying COS consumption in oxic soils is the destruction of COS by enzymes from the carbonic anhydrase family (Conrad 1996; Meredith et al. 2018; Ogawa et al. 2016; Seibt et al. 2006; Wingate et al. 2008), nitrogenase (Seefeldt et al. 1995), CO dehydrogenase (Ensign, 1995) and CS_2_ hydrolase (Smeulders et al. 2013). While COS uptake seems to outweigh COS emission in oxic soils in most cases, some evidence suggests that under dry, hot, or high-light conditions processes that emit COS can turn soil into a net source for COS (Kitz et al. 2017; Maseyk et al. 2014). The mechanisms underlying emissions from oxic soils are not very well understood and are thought to be a combination of abiotic and biotic processes. Among the abiotic factors, soil temperature (Meredith et al. 2018; Whelan and Rhew 2015), soil moisture (Bunk et al. 2017; Kaisermann et al. 2018) and light level (Kitz et al. 2020; Whelan and Rhew 2015) are considered to have the biggest impact on soil COS emission.

One component of the soil COS flux neglected so far is the impact of roots. At this point, we are not aware of any published study which would have investigated whether and how the presence of living plant roots affects the soil COS exchange. Two studies which measured the COS exchange of roots not connected to living plants are Maseyk et al. (2014), showing consistent COS emission from roots of multiple species of an agricultural field, and Whelan and Rhew (2015), observing increased COS emission from roots mixed with soil when exposed to light. But roots also contain the COS-consuming enzyme CA (Demir et al. 2009; Dimou et al. 2009; Yu et al. 2007) and are able to take up gases, like CO_2_ (Milton et al. 1998; Ota and Tanaka 2019; Rao and Wu 2017), from the surrounding soil, which could lead to COS uptake by living roots. In addition, plants can influence the surrounding soil and as a consequence alter gas exchange, be it biotic or abiotic in nature, from the soil. While in situ measurements, from ecosystem-scale eddy covariance to chamber measurements, of the COS exchange in many cases, inevitably include the root contribution and plant-soil interactions, lab experiments usually use soil samples uncoupled from the plants they are associated with in situ measurements. This separation of plant and soil might bias the data gained from those experiments, since for decades the scientific community is aware that plants influence the soil they grow in, primarily via their roots, which led to the creation of the term rhizosphere (Hiltner 1904). While there are different conceptual frameworks of the rhizosphere, its importance is undisputed (Canto et al. 2020; Philippot et al. 2013; York et al. 2016).

In this study we thus investigated the influence of living plant roots on the soil COS exchange, using young beech trees grown in potting soil under controlled laboratory conditions over the course of nine months. We hypothesized that.

(I) the soil COS flux would change when plant roots are present considering the multitude of metabolites released into the soil via root exudates.
(II) soil COS fluxes in root-influenced soil will exhibit a diurnal cycle following light availability and thus photosynthetic activity of the corresponding beech trees.
(III) the soil COS flux will change throughout the year, with environmental conditions in the lab kept constant, following changes in the phenological development of the investigated beech trees.

Answering these questions are a first step to bridge the gap between soil observations under laboratory conditions and field observations of root-influence soil.

## Materials and methods

### Trees and potting soil

18 young (~ two years old) beech trees (*Fagus sylvatica* L.) from a local tree nursery were root-washed and potted, with the same potting soil used later in the experiment, 7 months prior to the start of the experiment in October 2020. Average tree height in October 2020 was 32.8 cm and average maximum stem width was 8.4 cm (see Table S1 for more details including initial soil moisture). The potting soil (Hawita Fruhstorfer Erde – Aussaat- und Stecklingserde, HAWITA Gruppe GmbH, Germany) was peat-based with a pH of 5.9, a nitrogen content of 80 mg/l, a phosphorus content of 60 mg/l, a potassium content of 90 mg/l and a magnesium content of 100 mg/l.

The trees were grown in a greenhouse, which had only frost protection heating and was therefore considerably cooler in February. The lab temperature was set to the same value (25 °C) in all months, which meant that the soil in February would experience an unusually high temperature, especially in comparison to the temperature the soil experienced in the greenhouse, in which the pots were prepared in.

### Chamber design

**Fig. 1:**
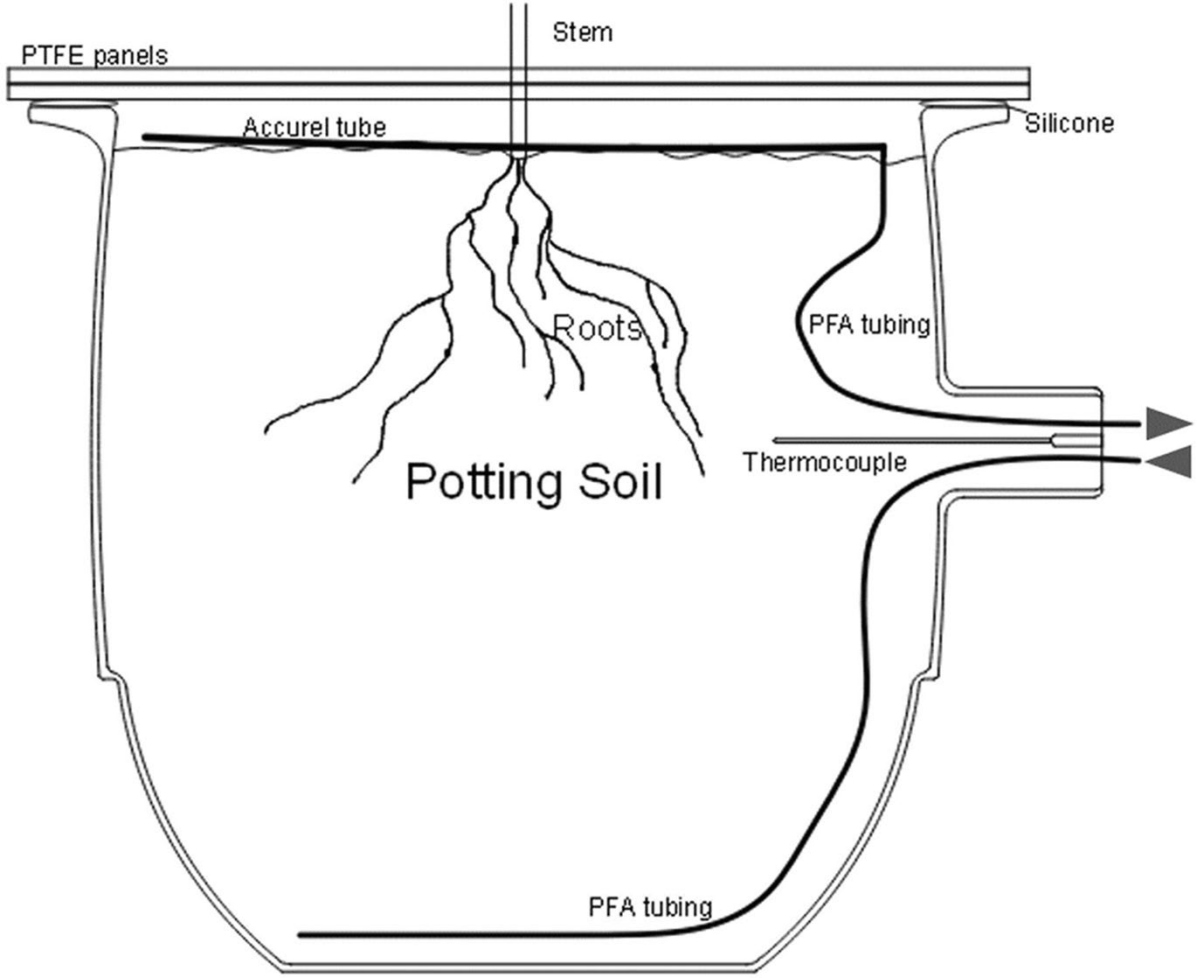
The chambers used in the experiment were desiccators with a multiport connector on the side, which allowed two PFA tubes (an inlet and an outlet) and one thermocouple to be inserted into the potting soil. The top PFA tube was connected to a semi-permeable Accurel tube. The top of the desiccator was sealed off using two PTFE panels and a silicone seal. Arrows indicate the direction of the air flow

**Fig. 2:**
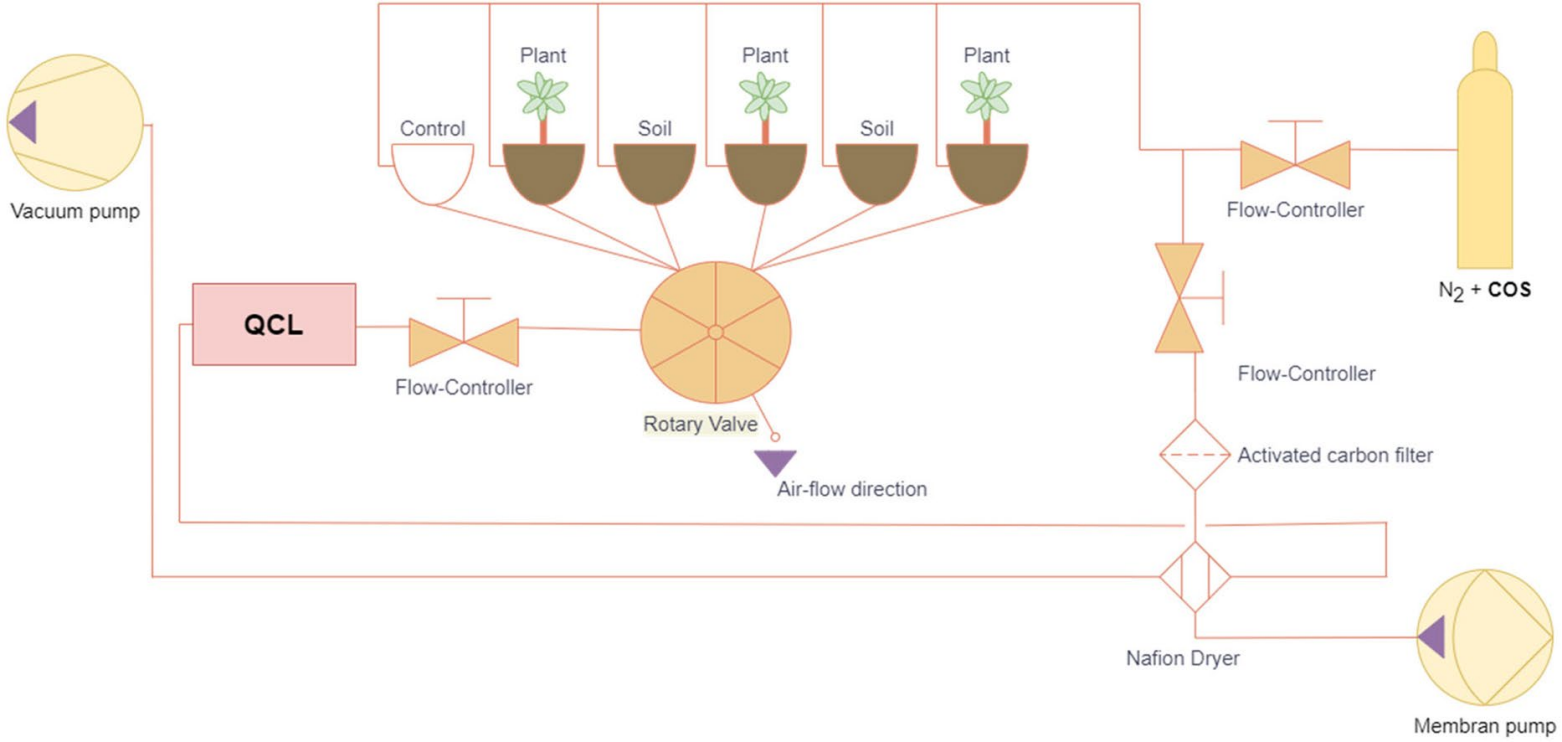
Schematic representation of the experimental setup. Purple arrows indicate airflow direction. The instrument measuring the COS and CO2 concentrations is denoted as QCL (Quantum cascade laser). Room air is entering the setup from the right-hand side (right-bottom) and is mixed with a known COS concentration from a gas bottle after all COS was removed from the room air stream using activated carbon filters. The resulting air mixture is supplied to the bottom of six chambers, one of them is measured, while the other five are flushed with the same flow rate

Six glass desiccators (Duran Mobilex with GL 32 thread, DN 200, DWK Life Sciences, Germany) with a volume of 5.8 l were used as chambers (see Fig. 1), three of them holding one beech tree each, two containing only potting soil and one control chamber without soil and/or plant (but otherwise identical to the treatment chambers). For each month a set of three new trees from the 18 trees in the greenhouse was used. Multiport connector caps (GL32, Duran) with a PTFE insert and four stainless steel tubing connectors were used to connect the PFA tubing with the chambers. Two layers of PTFE plates, with each layer consisting of two plates that could be connected and sealed via a tongue and groove system, were placed on top of the chamber with a hole in the middle for the stem of the beech tree (see Fig. 1). The PTFE plates together with a silicone ring closed off the inside of the chamber and allowed controlled circulation of the air through the PFA tubing. The airflow through the system was realized with two pumps, one pushing air through the system and one driving air to the instrument (see the sketch in Fig. 2). Room air was pushed through a nafion dryer (PD-100T-48MSS, Perma Pure, New Jersey, United States) and self-build charcoal filters, which have proven to be efficient in removing COS (Kesselmeier et al. 1999), towards a flow controller set to 2.175 l/min. A second flow controller set to 0.0085 l/min was connected to a gas cylinder containing a known COS concentration in nitrogen (Linde Gas GmbH, Stadl-Paura, Austria). Air with a concentration of 500 ppt was mixed by adjusting the flow controllers accordingly. The mixed air was pushed to the bottom of the soil column in the chambers at a flow rate of 0.36 l/min. Air-permeable, hydrophobic polypropylene tubes (Gut et al. 1998) (Accurel), arranged in a loop, on the top of the soil columns were used to sample air that has passed through the soil. The air was then either directed to the instrument measuring gas concentrations or to a common exhaust. The flow through all the chambers was therefore constant, with one being measured and the remaining five being flushed at any given time.

### Gas measurements

The Accurel tubes were connected to PFA tubing leading to a rotary valve (VICI 16 port valve, VICI Valco Instruments, Houston, USA), which was controlled by the instrument measuring the gas concentrations – an Aerodyne Dual QCL (Aerodyne Research, Billerica, MA, USA). The Aerodyne software TDLWintel controlled the rotary valve and calculated the COS and CO_2_ concentrations in real-time. The optical bench of the instrument was heated to a temperature of 35 °C and the absorption spectra were fitted at a frequency of 1 Hz. Air from one selected position on the rotary valve was pushed through another flow controller set to 0.36 l/min to the laser, while the rest was pushed to a common exhaust. In addition to pushing the air through the valve, a vacuum pump was sucking the air to the laser. To avoid pressure oscillations, the pressure lock was deactivated in TDLWintel and the cell pressure (~ 4 kPa) was adjusted manually at the beginning of the experiment by setting a fixed valve position and flow rate to the instrument. To test for leaks, the instrument including the vacuum pump was disconnected from the setup, and the flow at the third flow controller, which was directly in front of the Dual-QCL, was checked before each run of the experiment. In addition, a manometer was connected to the empty chamber to check if the overpressure in the system was constant throughout the experiment.

### Environmental variable measurements

During the measurements, the chambers were placed on self-build balances using 10 kg load cells (B6N-C4-10 kg-1B6, Zemic Europe B.V., Etten-Leur, Netherlands) and connected to a datalogger (CR5000, Campbell Scientific, Logan, US) to monitor weight loss, due to water loss via plant transpiration, by the chambers. The aboveground part of the trees received light from growth lamps (ViparSpectra 600 W Reflector LED with both modes switched on) for 10 h a day, the light intensity (PAR) at the top of the trees was approximately 1500 µmol m^−2^ s^−1^. A stainless-steel thermal sensor Typ K (RS Pro) with a rod length of 150 mm connected to a datalogger (CR5000, Campbell Scientific, Logan, United States) was used to measure the temperature inside the chambers.

### COS flux measurement

Measurements were carried out in 2021, with a set of measurements in February, April, May, June, and October. One week before the start of each measurement, three young beech trees were moved from their pots, with as little potting soil clinging to the roots as possible without severe disturbance of the roots, to the experimental chambers described above. The remaining volume was filled with potting soil, whereby a new package of potting soil was used for each month to avoid colonization by microbes once the steamed potting soil bags were opened. The same potting soil was used for the soil only chambers. After repotting all experimental chambers, the ones with trees and the ones with only potting soil, they were watered with 1 l of tap water.

For each measurement, one empty (control), two soil only, and three tree-containing (soil + root) chambers were used. In each half-hourly measurement cycle, every soil only and tree chamber was measured for three minutes preceded and followed by measurements of the control chamber. The temperature in the lab was set to 25 °C in all months.

After three days of measurements, leaves were harvested and scanned to get the tree leaf area and roots washed, dried and weighed to gain the root dry weight. The leaf area was used as an indicator for the phenological stages of the trees. The potting soil was sampled before and after the three-day measurement period, the soil samples were dried and weighed to obtain the fresh and dry weight, with which the gravimetric soil moisture was calculated, at the start and end of the experiment.

### Flux calculation and statistics

Fluxes were calculated using Python 3.7.13, IPython 7.22.0, Spyder 4.1.3, and the pandas library (McKinney 2010). Errors in air temperature, air pressure, flow rate and weight were propagated through flux calculations via the “uncertainties” package (Lebigot n.d.). The COS and CO_2_ fluxes were calculated according to Eq. 1.

1 $$F=\frac{q(Ce-Cc)}{DW}.$$

  

F is the soil flux in pmol kg^−2^ s^−1^ for COS and µmol kg^−2^ s^−1^ for CO_2_, q is the flow rate (mol s^−1^), Ce is the mean concentration in the control (empty) chamber before and after the measurement of the current chamber, Cc is the mean concentration in the current chamber and DW is the dry weight in kilograms (kg) of the potting soil.

Statistical analyses were carried out using the software R 4.2.2 (R Core Team 2018) and RStudio (RStudio Team 2016) and the following packages: data.table (Dowle and Srinivasan 2022), ggplot2 (Wickham 2016), lme4 (Bates et al. 2022), relaimpo (Groemping 2021), randomForest (Liaw and Wiener 2002), car (Fox et al. 2022), tsutils (Kourentzes 2022), stats (R Core Team 2018 ) and permimp (Debeer et al. 2021). No soil temperature data was available for the soil only chambers in May due to a thermocouple failure. No outliers were removed from the dataset. To test for significant differences between groups, Kruskal-Wallis rank sum test were performed. The flux data was decomposed, to separately investigate the daily variations and the 3-day trend, with the decompose function from the stats package using a moving average and a frequency of 24 h for the seasonal component (Suppl. Fig. 2). To characterize the temporal pattern of the daily variation and trend k-Means clustering was performed using the kmeans function from the stats package, providing the function with a value of three for the number of centers and 100 for the number of random sets (nstart). The relative importance of the predictors in the linear models were calculated using the boot.relimp function in the relaimpo package with b = 10,000, type = c(“lmg”, “last”), rank = TRUE, diff = TRUE and rela = TRUE. The relative importance values presented in the paper are the ones yielded by the lmg (Lindemann, Merenda and Gold) method (the “last” method did not yield a different ordering, see Suppl. Fig. 1). In order to calculate the root-only COS fluxes the means of the soil only chambers for each month were calculated and subtracted from each beech tree measurement. In order to investigate the variable importance of the root-only COS data, a random forest regression with 1000 trees was performed followed by an analysis for retrieving the conditional permutation importance (permimp function).

## Results

**Fig. 3:**
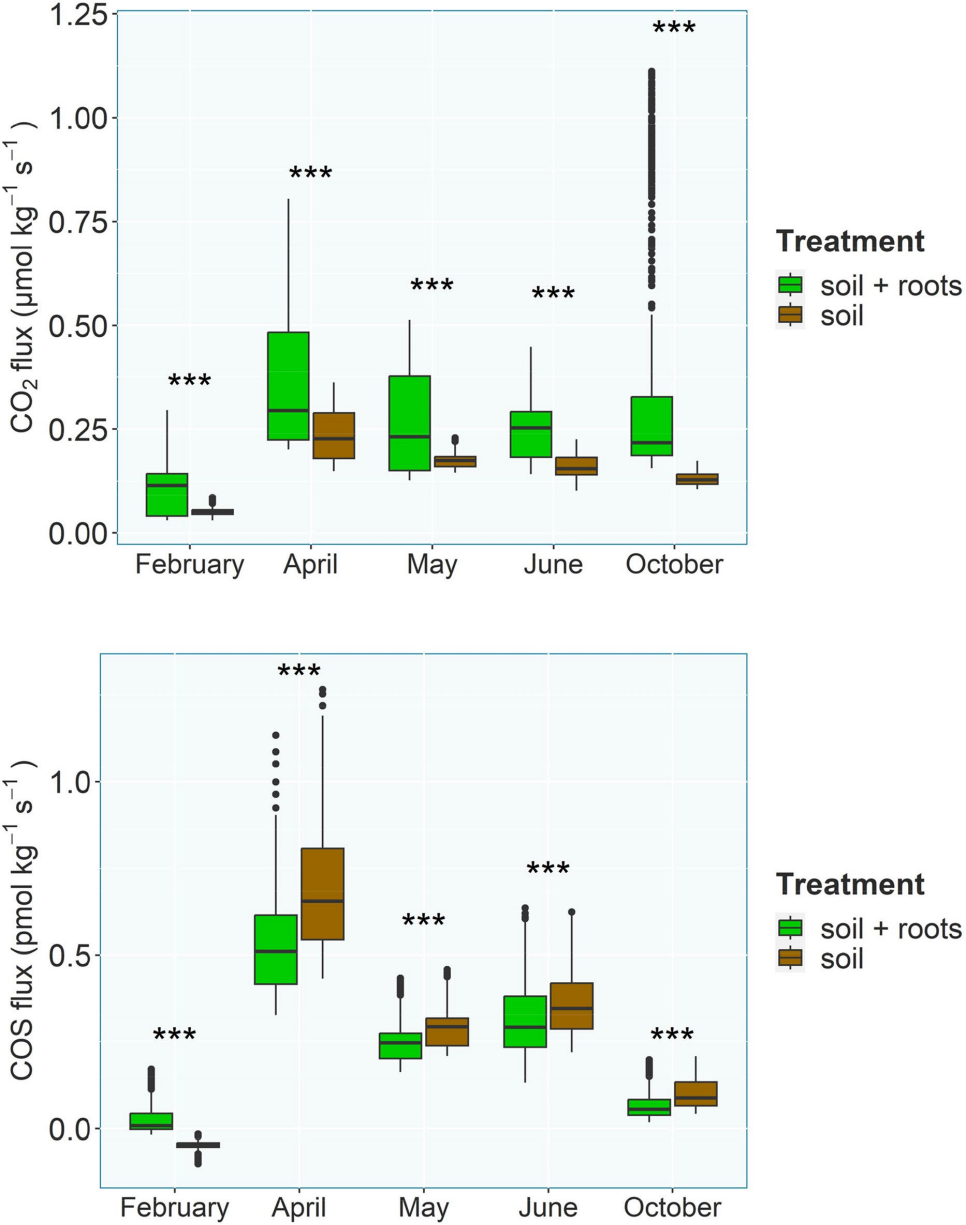
Flux observations of CO_2_ (top panel) and COS (bottom panel) for chambers containing either soil alone or soil and a beech tree as boxplots for each month. Asterisks indicate significant differences (***: *p* < 0.001) between the two treatments using Kruskal-Wallis rank sum tests

**Fig. 4:**
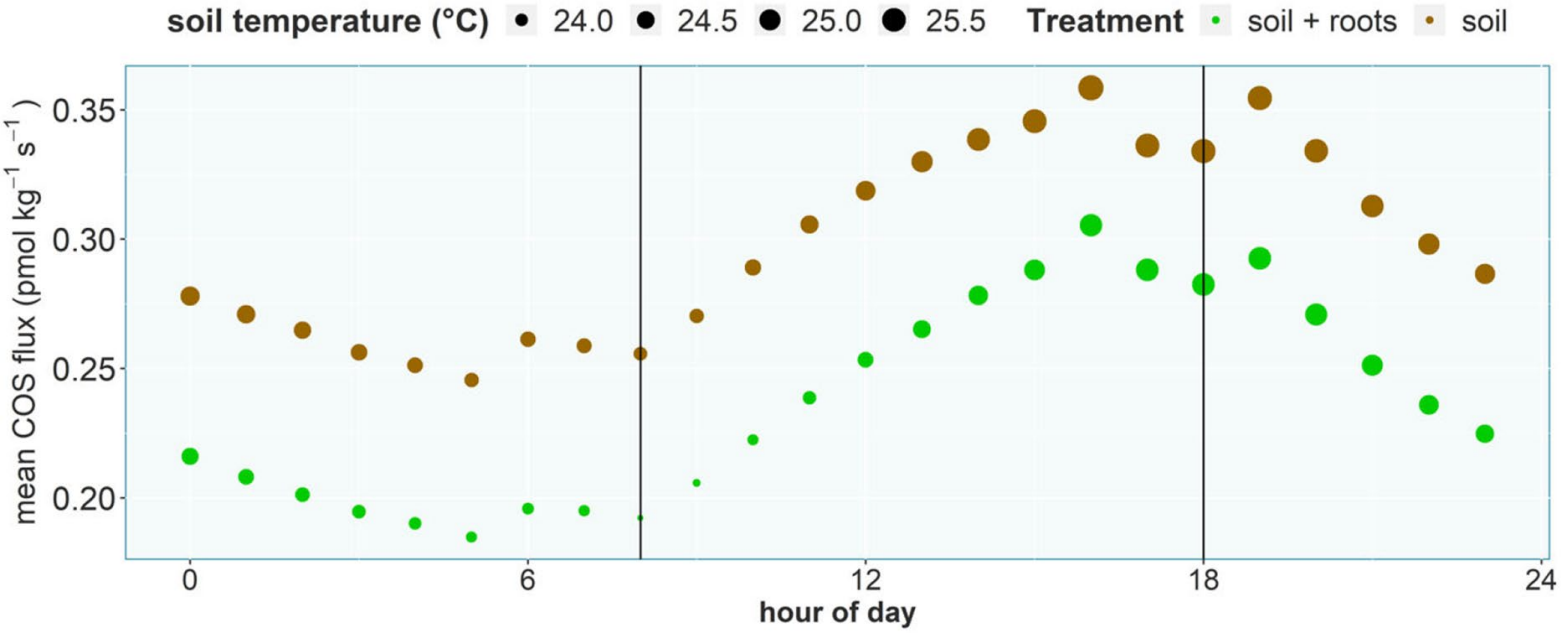
Mean diurnal cycle of COS fluxes across all experiments. Point size indicates the mean soil temperature. Black lines show start (8:00) and end (18:00) of light exposure

Mean soil COS emission, CO_2_ emission, and soil temperature were highest in April for both soil (COS: 0.693 pmol kg^−1^ s^−1^, CO_2_: 0.233 µmol kg^−1^ s^−1^, 26.8 °C) and soil + root (COS: 0.534 pmol k^−1^ s^−1^, CO_2_: 0.372 µmol kg^−1^ s^−1^, 27.0 °C) and lowest in February, again for both soil (COS: 0.047 pmol kg^−1^ s^−1^, CO_2_: 0.050 µmol kg^−1^ s^−1^, 21.5 °C) and soil + root (COS: 0.026 pmol kg^−1^ s^−1^, CO_2_: 0.108 µmol kg^−1^ s^−1^, 21.8 °C). For each month, the differences between soil and soil + root for COS and CO_2_ were highly significant (Kruskal-Wallis rank sum test – *p* < 2.2e^−16^), with higher CO_2_ and lower COS emissions in the soil + root chambers (except for February) (Fig. 3). COS fluxes were moderately to highly (R^2^ > 0.3) correlated to soil temperature, except for the soil treatment in April (see Table 1; Fig. 4). The COS to CO_2_ flux ratio was higher in the soil treatment compared to the root treatment, that is to say, the presence of roots reduced the emission of COS per unit of emitted CO_2_, except in February. The soil moisture decrease from the start to the end of each 3-day experiment as a mean over all months was 1.65% in the beech chambers and 0.54% in the soil chambers. The mean soil moisture decrease over both treatments was highest (2.83%) in June and lowest in October (0.48%).

**Table 1:** Pearson correlation coefficients for both treatments (soil and soil + root) between the COS flux and the soil temperature (no correlation in May since the soil temperature data are missing)

	Pearson correlation coefficient	
	Soil	Soil + Root
February	0.77	0.35
April	-0.11	0.64
May	-	0.58
June	0.74	0.61
October	0.91	0.91

**Fig. 5:**
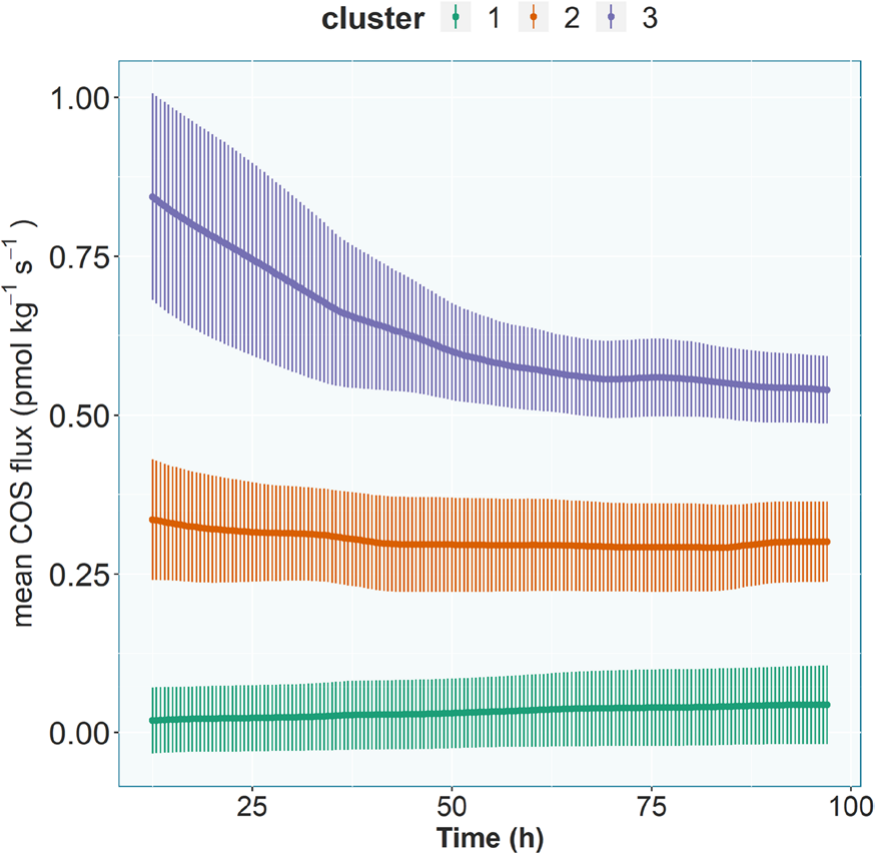
The mean (+ sd) COS temporal change for each of the three clusters created by kmeans clustering. Cluster 1: February, October; Cluster 2: April (one beech replicate), June, May; Cluster 3: April (remaining replicates)). The first cluster contains the pre-season and late season measurements, while the second cluster contains measurements taken during the peak growing season (except for one replicate in April, which was assigned to its own cluster)

The change of the COS flux over the course of one experiment (henceforth referred to as temporal change) varied between months, but could be organized, using kmeans clustering, into three distinct groups (Fig. 5). In April there was an overall decrease in the COS flux over the course of the experiment, with a steep decline at the beginning and only marginal changes towards the end of the measurements. In June and May the slope of the COS flux trend was close to zero (Fig. 5). In February and October, the COS flux increased slightly throughout the experiment. The kmeans clustering did only group the COS flux trends by month and did not separate the different treatments from each other.

The “periodic” component with a frequency of 24 h (from now on called “daily component” or just “daily” in variable names) was also clustered using kmeans. Group three contained the daily components of most replicates in April and two of the replicates in October and had a mean amplitude of 0.091 pmol kg^−1^ s^−1^. Group two contained the daily components from June with an amplitude of 0.114 pmol kg^−1^ s^−1^. The daily components of the remaining replicates were clustered together in group three with an amplitude of 0.024 pmol kg^−1^ s^−1^ (Suppl. Fig. 3).

**Fig. 6:**
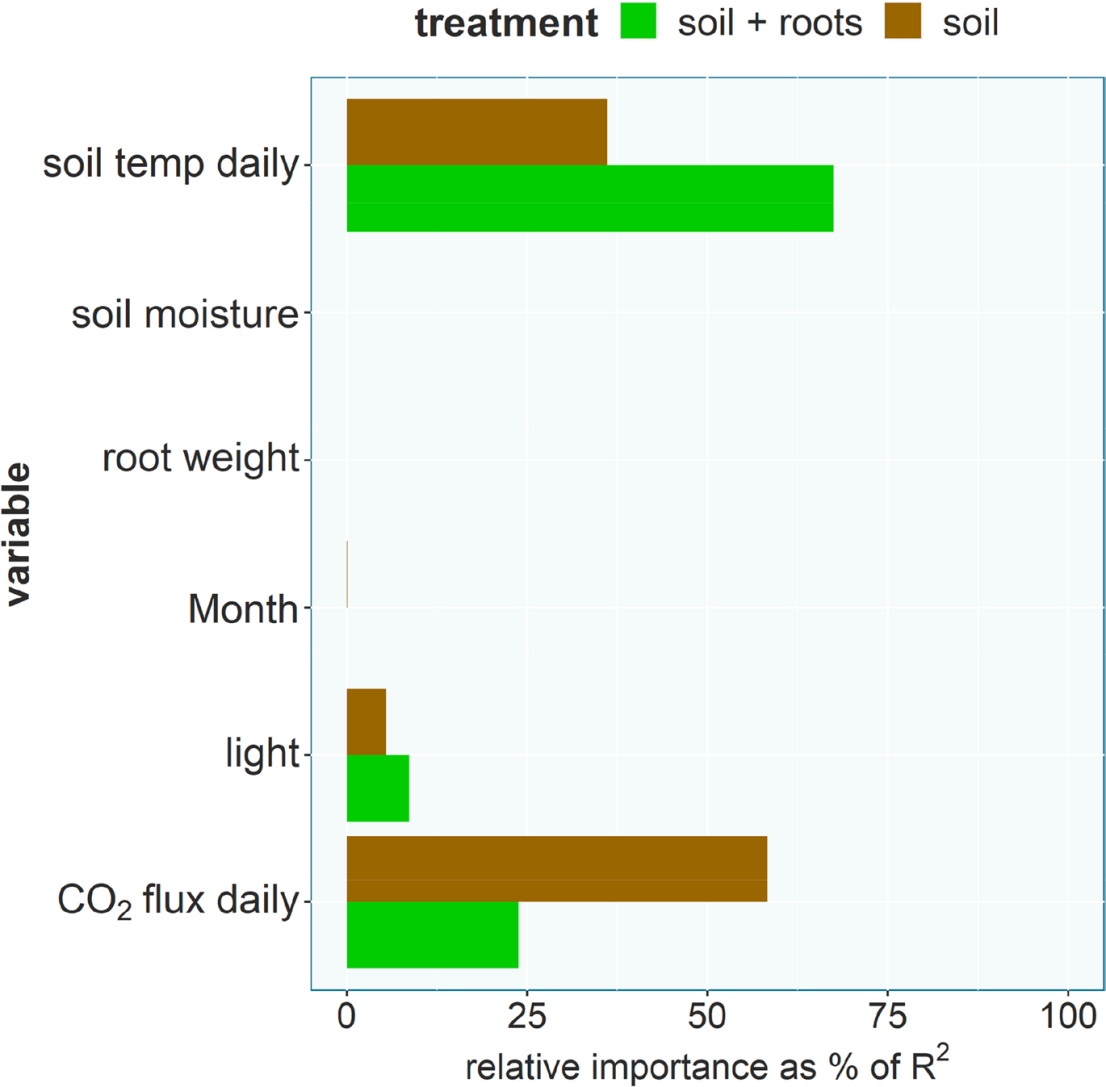
Relative variable importance for the predictors in the two linear models using the daily component data from either the soil (R^2^: 83%) or soil + root (R^2^: 78%) treatment

Two multiple linear regressions were performed on the daily component data, one for each of the two treatments. For the multiple linear regressions COS-daily-soil, $cosfl\widehat{ux}dailysoil={\widehat\beta}_0+{\widehat\beta}_1soiltemperaturedaily+{\widehat\beta}_2CO2fluxdaily+{\widehat\beta}_3light+{\widehat\beta}_4rootweight+{\widehat\beta}_5soilmoisture+{\widehat\beta}_6month$, and COS-daily-root, ${cosfl\widehat{ux}dailyroot}=\widehat{\beta}_{0}+\widehat{\beta}_{1}soiltemperaturedaily+\widehat{\beta}_{2}CO2fluxdaily+\widehat{\beta}_{3}light+\widehat{\beta}_{4}rootweight+\widehat{\beta}_{5 }soilmoisture+\widehat{\beta}_{6} month$, no multicollinearity (Variance inflation factors < 4) were detected and both models had the predictive capability (F Statistic: *p* < 0.05). The COS-daily-soil model explained 83% of the variability in the response variable (COS flux daily soil) and all predictors were highly significant (*p* < 0.001). The most important predictor in the model was the daily component of the CO_2_ flux (CO_2_ flux daily) followed by daily soil temperature and light (Fig. 6). The COS-daily-root model explained 78% of the variability in the response variable (COS flux daily root) with all predictors except the root weight being highly significant. The most important predictor in the model was the daily component of the soil temperature followed by the CO_2_ flux and light (Fig. 6).

**Fig. 7:**
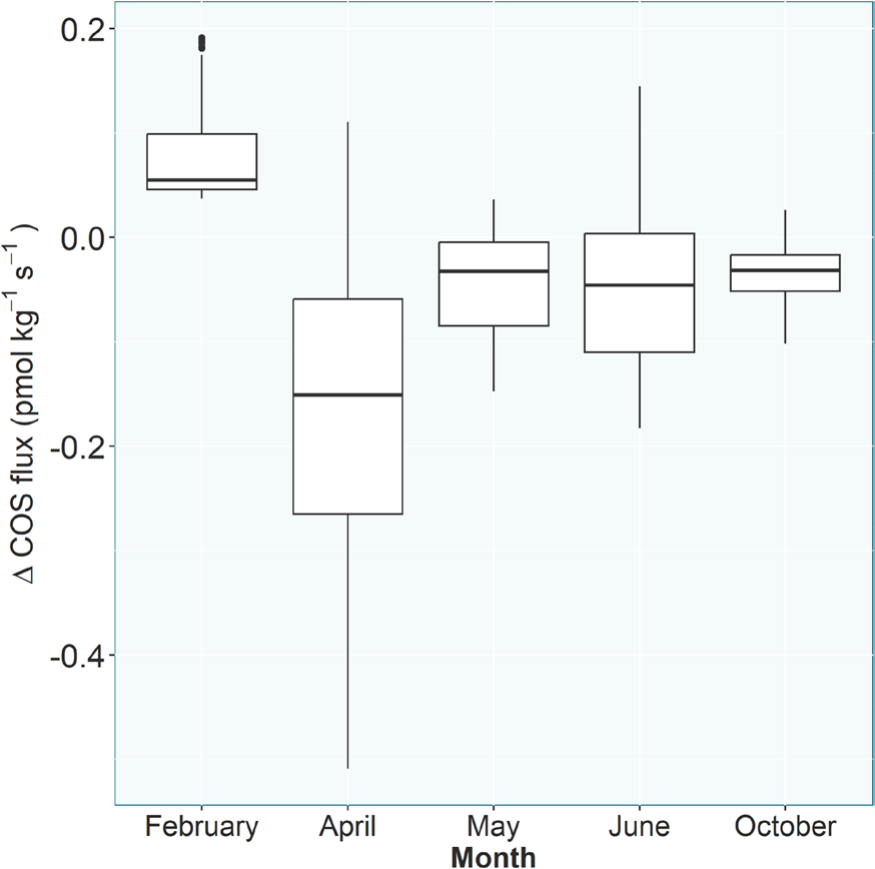
Root-only soil COS fluxes derived by substracting the COS flux from the soil only chambers from the ones containing beech trees

**Fig. 8:**
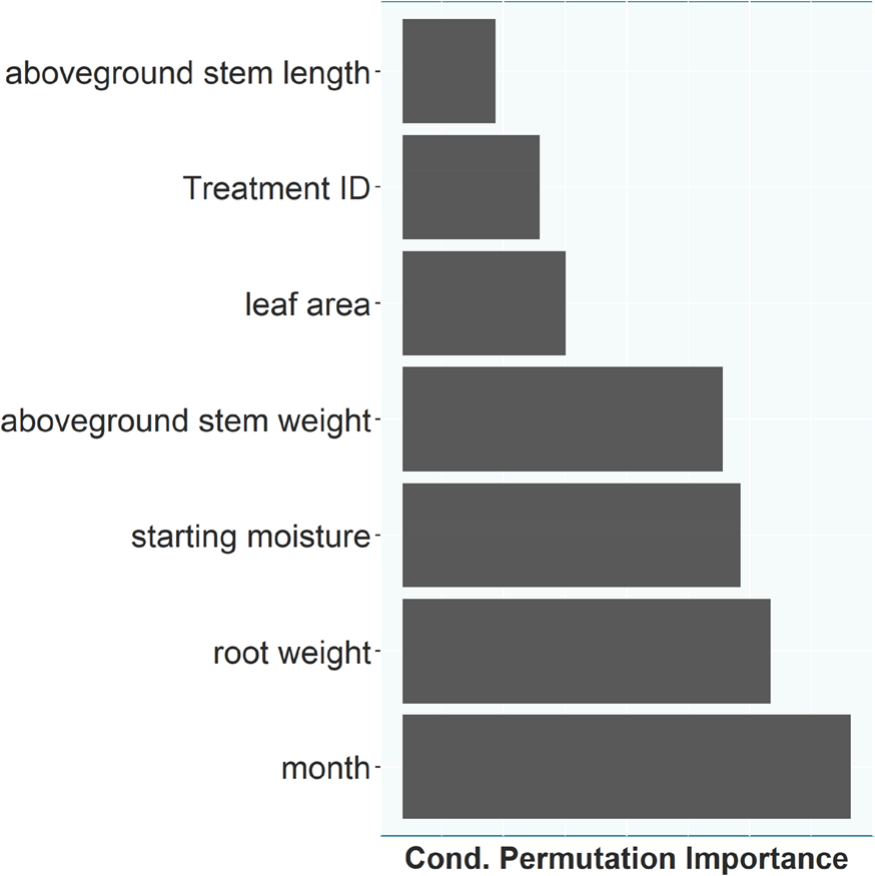
Conditional permutation importance in the random forest regression using the root-only soil COS flux data. Month is the most important variable in the random forest to predict the observed COS flux followed by root weight

The mean differences in the COS flux between the soil + root and soil treatments were 0.074 pmol kg^−1^ s^−1^ in February, -0.159 pmol kg^−1^ s^−1^ in April, -0.041 pmol kg^−1^ s^−1^ in May, -0.046 pmol kg^−1^ s^−1^ in June and − 0.036 pmol kg^−1^ s^−1^ in October. The mean difference in the CO_2_ flux was 0.058 µmol kg^−1^ s^−1^ in February, 0.139 µmol kg^−1^ s^−1^ in April, 0.086 µmol kg^−1^ s^−1^ in May, 0.093 µmol kg^−1^ s^−1^ in June and 0.217 µmol kg^−1^ s^−1^ in October (Fig. 7). The random forest regression performed on the COS difference data (root-only) as response and the time series independent variables (root weight, leaf area, starting soil moisture, month, stem length, stem weight, tree ID) as predictors explained 79.06% of the variance (out of the bag). The conditional permutation importance ranked month as the most important predictor closely followed by root weight and initial soil moisture (Fig. 8).

## Discussion

Throughout most of the experiment (except in February), both treatments were a source of COS, which is in contrast to many previous lab and field studies (see Whelan et al. (2018) and Whelan et al. (2022) characterizing oxic soils as a COS sink. This is most likely due to the very nutrient rich potting soil used in the experiment, which is commonly used as a cultivation soil in gardening and cannot be compared to natural soils. The choice to use an “artificial soil” was made to provide an as much as possible homogeneous substrate across all replicates and throughout the duration of the experiment in order to focus on the root contribution and to use the same soil for the cultivation of the trees and the experiment itself. During the growing season, COS fluxes in chambers containing beech trees were significantly lower compared to the chambers containing only soil (Fig. 3), while higher CO_2_ emissions from those chambers indicate increased root and soil respiration. COS fluxes in February showed the reverse, with higher COS fluxes from the soil + roots chambers (Fig. 3). One explanation for the lower COS fluxes in chambers with higher biological activity, as indicated by increased CO_2_ emission, is the biotic uptake of COS by the ubiquitous enzymes of the carbonic anhydrase family, which have been shown to consume COS in microorganisms (Masaki et al. 2016; Ogawa et al. 2013, 2016) and can also be found in roots (Demir et al. 2009).

Since soil respiration was higher in the beech chambers in February compared to the soil chambers (Fig. 3), overall decreased activity and the resulting decrease in the transfer of metabolites to the soil, which could be hypothesized due to the missing leaves and therefore inability of the plant to photosynthesize, cannot be the sole explanation for the reverse behavior in February compared to the rest of the year. One reason might be differences in the microbial community composition. Izumi (2018) showed changes in rhizosphere species composition after the defoliation of birch and pine trees, which could lead to changes in COS emission and uptake behavior (Behrendt et al. 2019; Kitz et al. 2019; Meredith et al. 2019). A major process by which plants influence the soil is rhizodeposition (Haichar et al. 2014; Jones et al. 2009), a term encompassing root exudates, mucilage, border cells, and gases released by plant roots. The investment into the rhizosphere by plants is substantial, with an estimated 5–21% of the total carbon fixed by photosynthesis ending up being released into the rhizosphere via root exudates (Derrien et al. 2004; Lynch and Whipps 1990; Marschner et al. 2008; Nguyen 2003). Not only the quantity, but also the composition of root exudates varies greatly, including amino acids, sugars, enzymes, organic acids, fatty acids, sterols, growth factors, vitamins, flavonoids, alcohols, and nucleotides/purines (Dennis et al. 2010; Vives-Peris et al. 2020). Quantity and quality of root exudates not only depend on the species, but also on the time of the day (Hubbard et al. 2018; Staley et al. 2017), age, and external biotic and abiotic factors (Jones et al. 2004). Changes in the quality and quantity of rhizodeposition providing microorganisms with a different substrate could therefor change the COS flux (Behrendt et al. 2019; Conrad 1996). For example, compounds known to be involved in COS emission are thiocyanates (Conrad 1996), which can be exuded by roots (Halkier and Gershenzon 2006; Xu et al. 2017). But rhizodeposition during the off-season is poorly understood even though plant root activity during that period does not cease completely (Andresen and Michelsen 2005; Kaiser et al. 2010). The observed difference between root-influenced and pure soil in this experiment was primarily an offset, while the trend and diurnal cycle were not substantially different (see Figs. 4 and 5). Nevertheless, hypotheses I – a change in the COS flux, when roots are present – cannot be rejected by this experiment.

The observed diurnal cycle in COS exchange was primarily driven by other abiotic factors rather than light availability, which we hypothesized (hypothesis II) would influence the COS fluxes of the corresponding soil via photosynthetic activity of the plant. Soil temperature, which was identified in previous studies (Kitz et al. 2017; Meredith et al. 2018; Whelan and Rhew 2015) to be a decisive factor for the soil COS flux, especially COS emission, was the most important predictor for the diurnal variation in the soil COS flux (Fig. 6). While soil moisture, which linearly decreased over the duration of each measurement, due to the slow drying of the soil, since no water was added after the start of each experiment, was an important predictor in the random forest regression to explain differences in the root-only COS fluxes (Fig. 8). The COS flux trends further reveal that the faster drying by the soil due to transpiration by the beech trees did not affect the COS flux trend in comparison with the soil only chambers, even though soil moisture dropped faster and to a lower overall value at the end of the experiment. Previous lab studies (Bunk et al. 2017; Kaisermann et al. 2018; Whelan et al. 2016) have shown that soil moisture can have a strong impact on the soil COS flux, especially if the soil gets very dry. In this experiment the very organic potting soil was fairly moist and dried little over the course of the three-day measurements, resulting in a small range of observed soil moisture values, which most likely meant neither plant nor soil were water limited at any time. While both treatments were driven in a similar fashion by the abiotic drivers, the variable importance for the diurnal cycle was different, with COS fluxes in the soil + roots treatments primarily driven by soil temperature and COS fluxes in the soil only treatments primarily driven by biological activity. Even though those two variables, soil temperature and soil respiration, are closely linked, the observed difference might indicate a stronger contribution by soil microbiota, in the soil only chambers the only contributors to soil respiration, to the soil COS flux, in comparison to the direct root contribution. The lack of light as a significant predictor might be caused by a delayed impact on COS exchange, since the literature suggests a delayed response of root exudation to plant photosynthesis (Ekblad and Hogberg 2001; Nakayama and Tateno 2018), which could have further masked the response of the COS flux to soil temperature and plant light availability. The parallel diurnal cycle of the soil and the soil + roots treatments (Fig. 4) further supports the interpretation of a missing impact of plant light exposure to soil COS exchange, since the soil treatment was in opaque chambers and the potting soil therefore not exposed to light.

The differences in COS fluxes between the months from the soil + roots chambers throughout the year suggest a seasonal pattern, which confirms our third hypothesis, but the soil only chambers showed a strikingly similar seasonal pattern. For that reason, the root only component was calculated (Fig. 7), assuming COS uptake and emission would not change substantially with changing local COS concentrations in the soil pore space. The month of the experiment, a proxy for the different phenological stages of the beech trees, was still the second most important predictor in the random forest to differentiate between the root-only COS fluxes in the beech chambers. This suggests an impact by changing plant activity during the course of the season on the soil COS fluxes, especially earlier in the season, while the impact in summer and autumn was minimal in regard to the overall magnitude of the flux (Fig. 7). Differences in rhizodeposition quantity and quality throughout the season are well documented in the literature (Edwards et al. 2018; Kaiser et al. 2010; York et al. 2016). Plants change the allocation of their carbon throughout the year redistributing resources to meet their needs, which does not necessarily have to be recently fixed carbon (Klein and Hoch 2015). This ties into the dependence of the root-only COS flux on root weight (Fig. 7), which varies throughout the season, and is known to influence root exudate quantity, while the impact of root morphology, e.g. young vs. mature roots, identified by previous studies to influence root exudation quantity and composition (Iannucci et al. 2021; Proctor and He 2017), was not investigated in this study. COS flux trends throughout the year were similar between both treatments, indicating no clear impact of tree phenology on observed soil COS fluxes (Fig. 5). The observed difference in the COS flux trend between summer and spring/fall treatments might rather be due to the difference between greenhouse and lab temperature and as a consequence the soil temperature, which was largest in February, with an air temperature difference of approximately 15 °C. Possibly, the seasonal differences may also reflect associated changes in the microbial community composition (Devi and Yadava 2006; Edwards and Jefferies 2013).

## Conclusion

Roots, a part of the ecosystem neglected so far, have an impact on the soil COS flux. In our study the presence of live roots decreased soil COS emissions, the suggested mechanisms are direct COS uptake by the roots themselves or root-mediated changes in microbiological composition or activity. But while the presence of live roots changed the overall magnitude of the COS flux, they did not, at least in this experiment, change the response of the soil to environmental parameters, like temperature and soil moisture. We have furthermore not seen an impact of the plant circadian clock on the soil COS flux. Research carried out so far that did use soil samples without live roots may have missed the influence of roots on the soil COS flux and might therefore not accurately reflect the COS flux regime in situ. Future research should try to disentangle the direct root contribution from the root-mediated contribution of the rhizosphere. Our experimental setup may also provide useful information for exploring the relationship between soil heterotrophic respiration and COS exchange, incorporated in some land surface models. Additional studies, exploring different plant species and (natural) soil types, are needed to further our understanding of the complex interactions, which lead to the surface COS exchange.

### Supplementary information

Below is the link to the electronic supplementary material.

ESM 1 (DOCX 572 KB)

## Data Availability

The data used in the current study is available under the DOI 10.5281/zenodo.10021106.
