# Supplementary material for: Root and rhizosphere contribution to the net soil COS exchange
Source: Plant Soil. 2023 Dec 23;498(1-2):325–39. doi: 10.1007/s11104-023-06438-0 (PMC11039419; doi:10.1007/s11104-023-06438-0)
Supplement: Supplementary file 1 — (DOCX 572 KB) [file 11104_2023_6438_MOESM1_ESM.docx]

**Supplementary information for**

**Root and rhizosphere contribution to the net soil COS exchange**

Florian Kitz^1^, Herbert Wachter^1^, Felix Spielmann^1^, Albin Hammerle^1^ and Georg Wohlfahrt^1^

**Affiliations:**

^1^Universität Innsbruck, Institut für Ökologie, Innsbruck, Austria

**Corresponding author:**

Florian Kitz

Sternwartestraße 15, 6020 Innsbruck, Austria

e-mail: florian.kitz@uibk.ac.at

telephone number: +43 512 507 51646

**Table S1.** Stem length, LAI and root dry weight for each tree used in the experiment (ordered by month).

| Month | Tree - ID | Stem length (mm) | LAI | Root weight (g) | Starting soil moisture (%) |
| --- | --- | --- | --- | --- | --- |
| February |  |  |  |  |  |
|  | beech 1 | 235 | 0 | 1.711 | 72 |
|  | beech 2 | 315 | 0 | 2.807 | 72 |
|  | beech 3 | 280 | 0 | 3.931 | 72 |
| April |  |  |  |  |  |
|  | beech 1 | 245 | 565 | 7.233 | 74 |
|  | beech 2 | 300 | 304 | 4.274 | 76 |
|  | beech 3 | 295 | 308 | 11.482 | 75 |
| May |  |  |  |  |  |
|  | beech 1 | 365 | 801 | 5.978 | 78 |
|  | beech 2 | 240 | 214 | 4.97 | 78 |
|  | beech 3 | 410 | 677 | 4.84 | 77 |
| June |  |  |  |  |  |
|  | beech 1 | 340 | 560 | 13.178 | 77 |
|  | beech 2 | 325 | 449 | 7.164 | 75 |
|  | beech 3 | 385 | 410 | 9.162 | 75 |
| October |  |  |  |  |  |
|  | beech 1 | 255 | 244 | 6.219 | 74 |
|  | beech 2 | 303 | 491 | 16.007 | 74 |
|  | beech 3 | 310 | 400 | 11.723 | 73 |


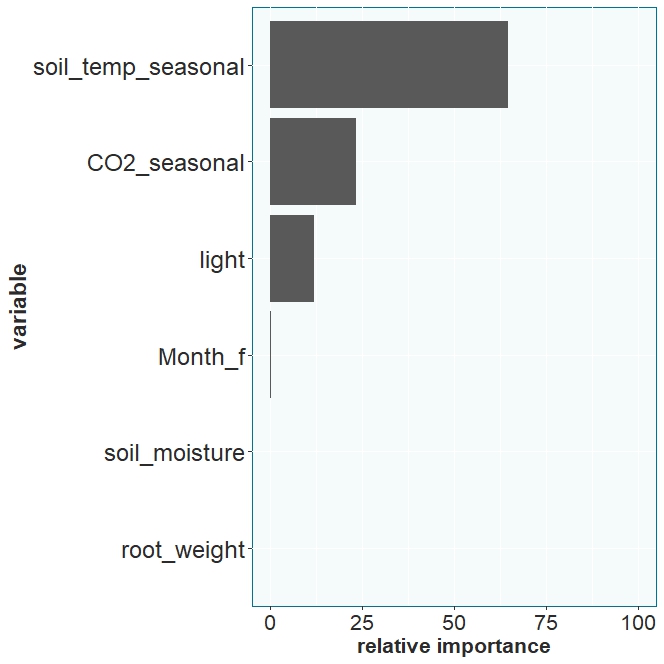


**Suppl. Fig. 1.** Relative variable importance of the linear model for the soil+root chambers using the “last” method in the relaimpo package.


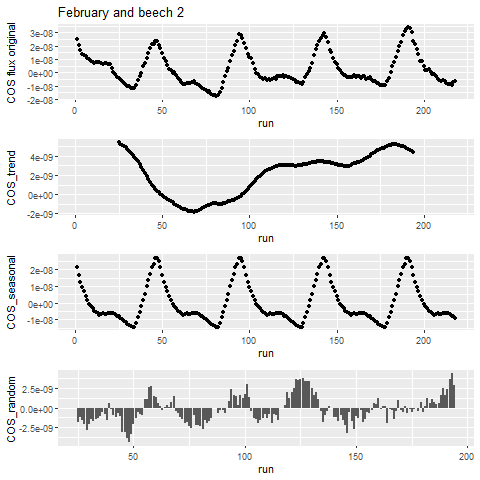

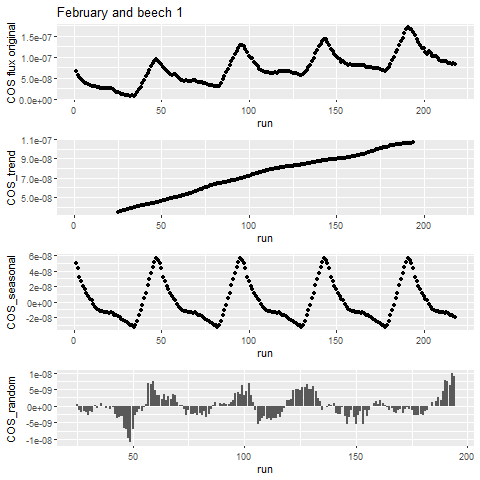


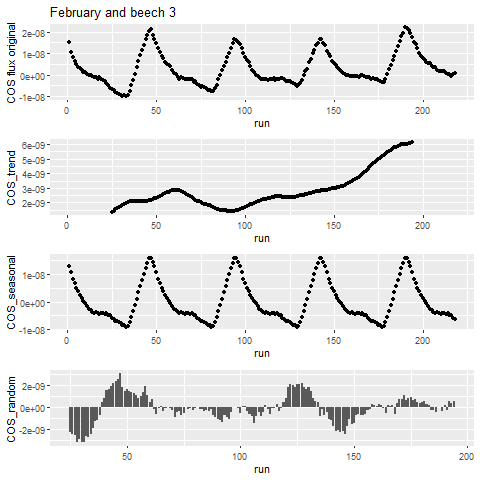

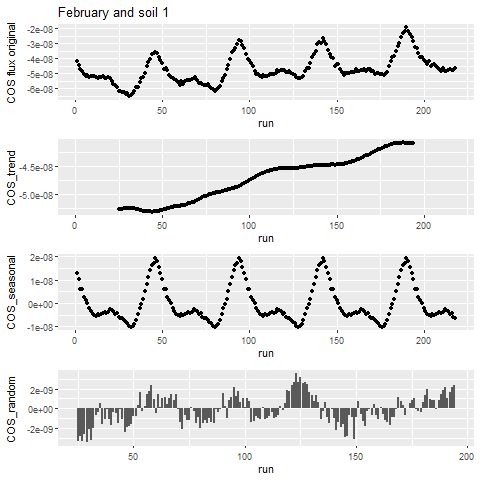

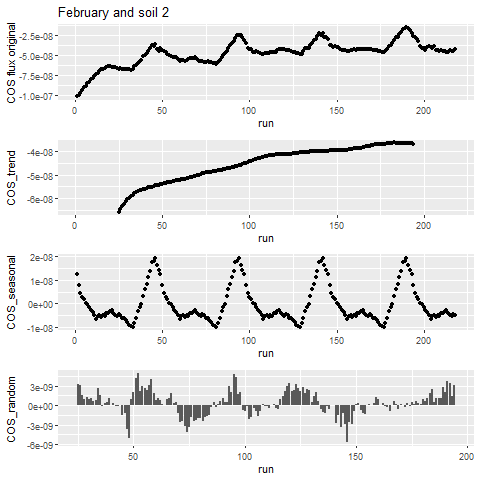

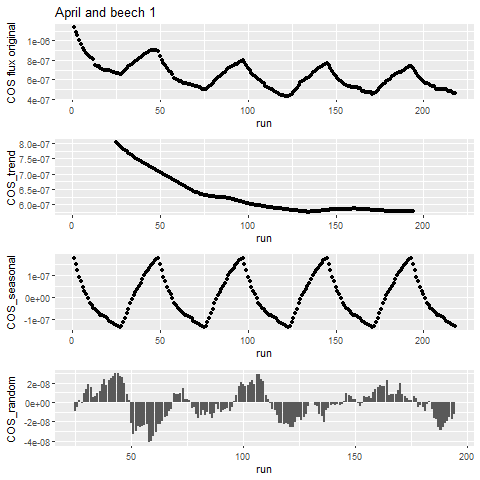

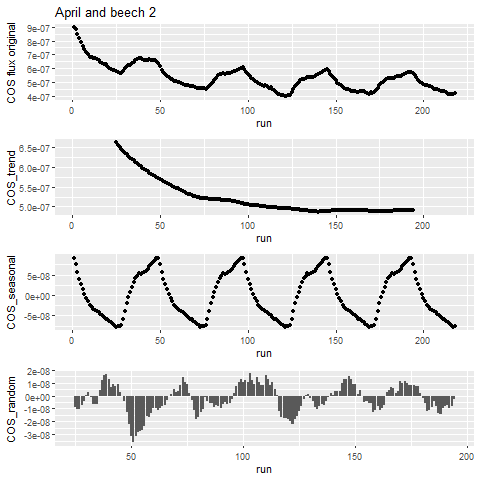

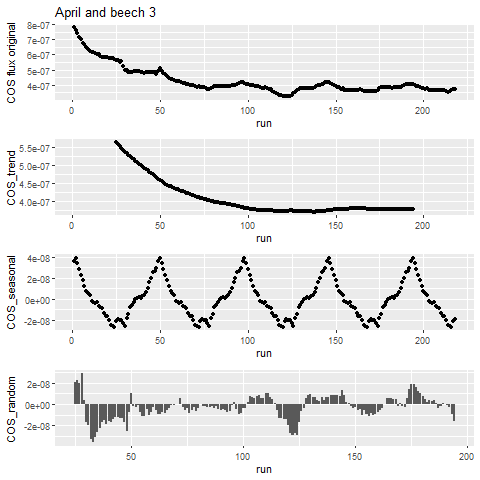

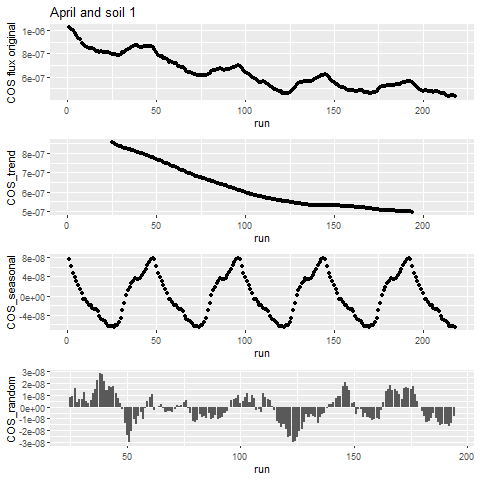

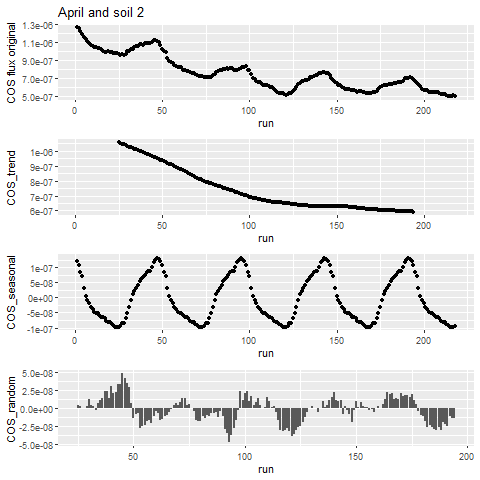

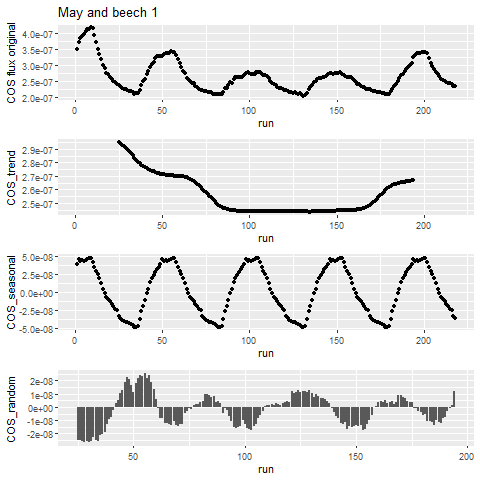

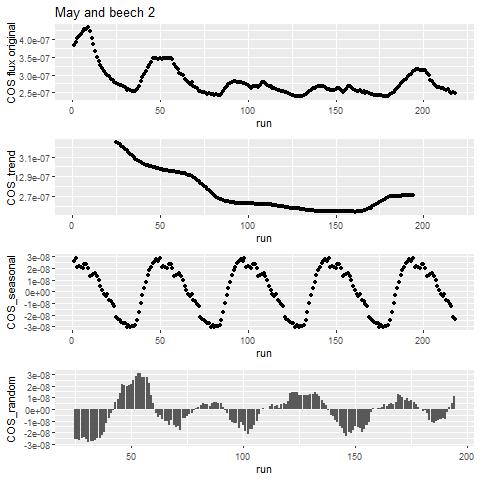

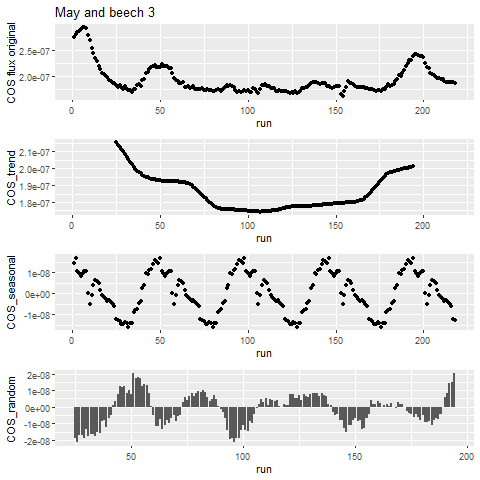

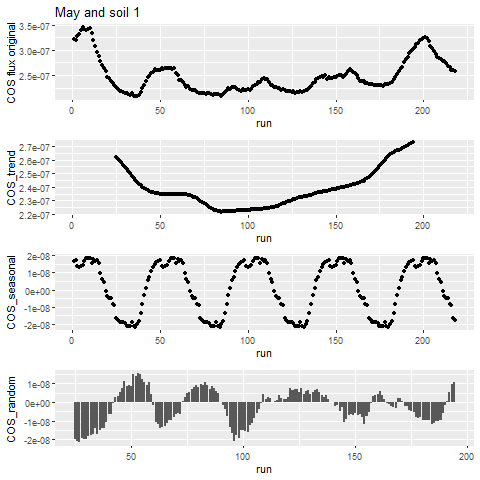

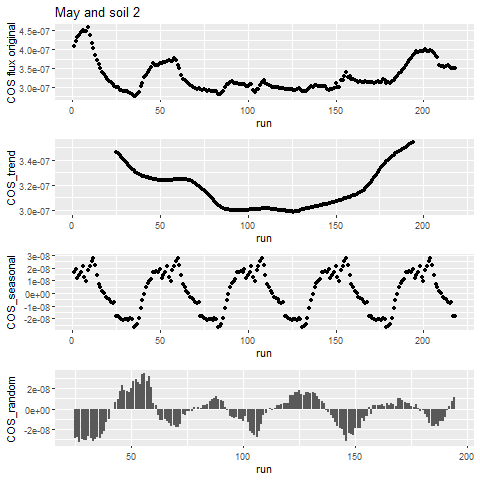

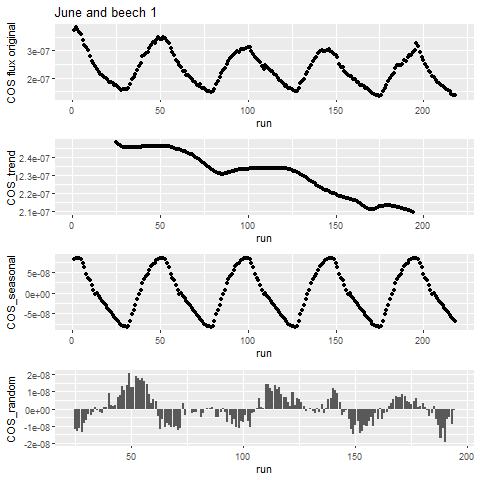

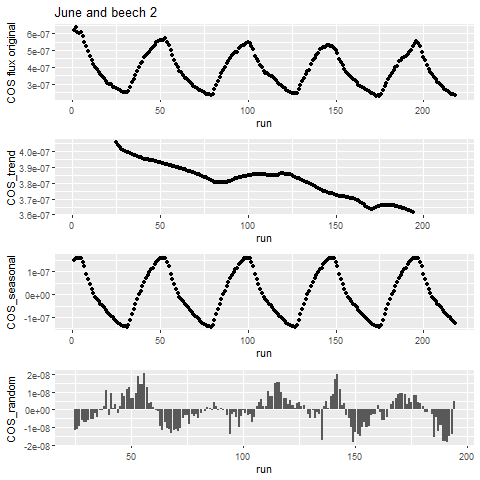

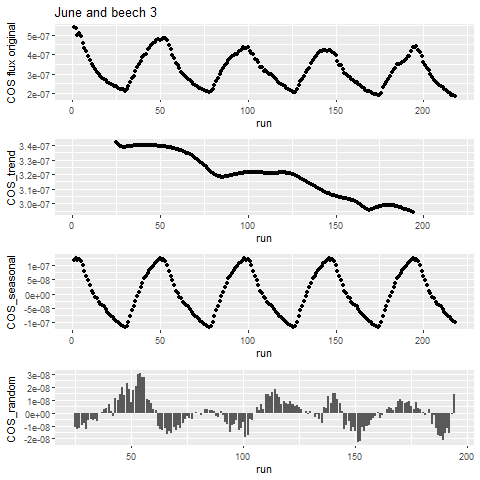

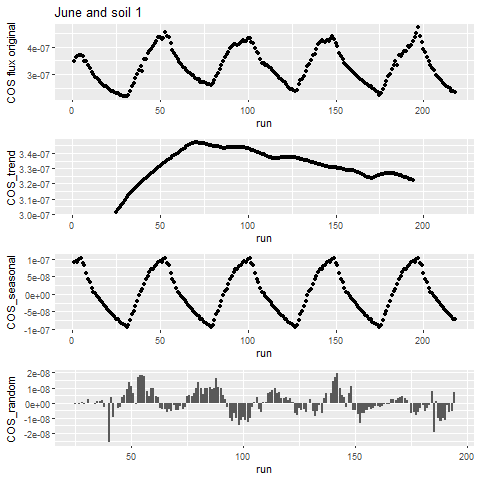

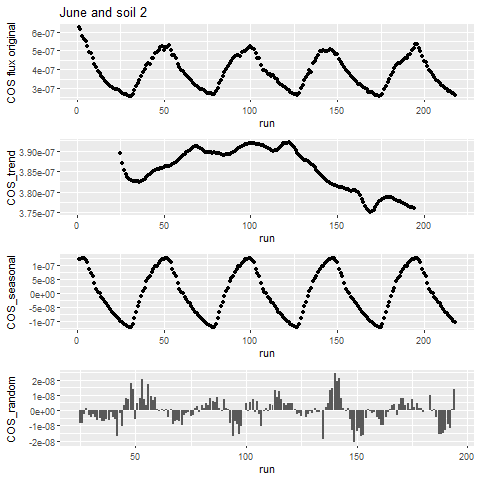

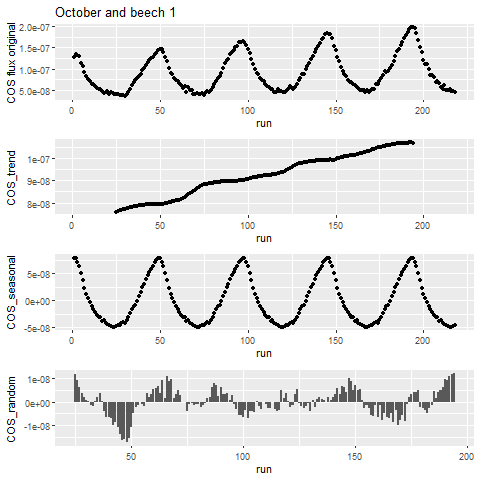

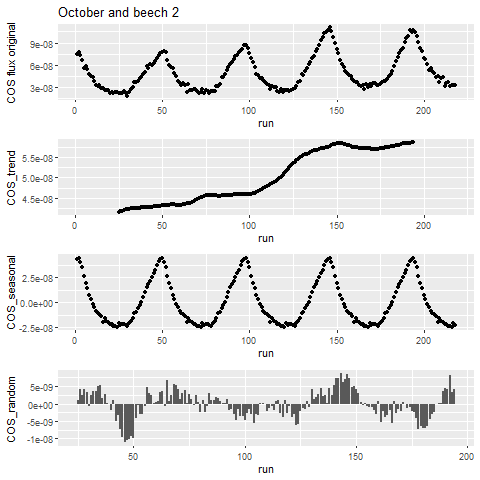

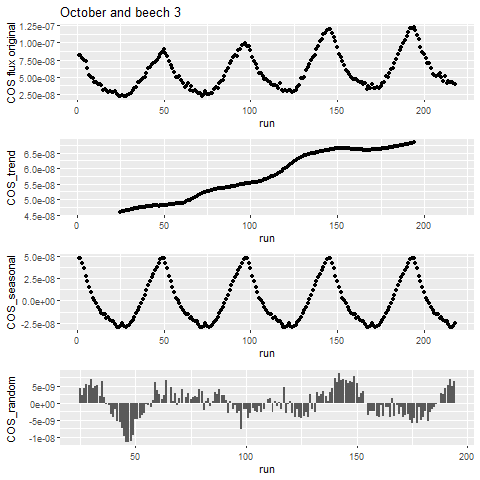


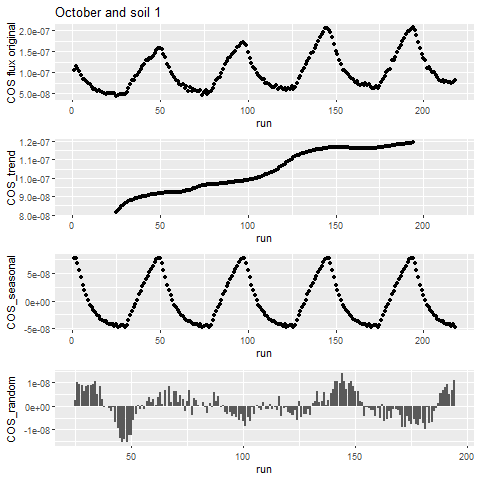


**Suppl.Figure 2** Timeseries data for each month and replicate. The first panel shows the original COS flux in pmol m^-2^ s^-1^, the second panel the trend component after the decomposition, the third panel the “seasonal” (or daily) component and the fourth panel the random component.


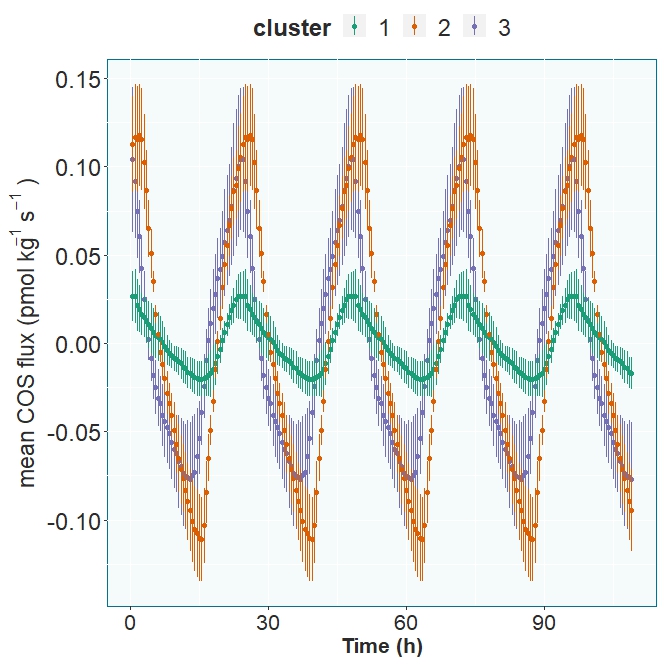


**Suppl. Figure 3** The mean (+sd) COS daily component for each of the three clusters created by kmeans clustering.


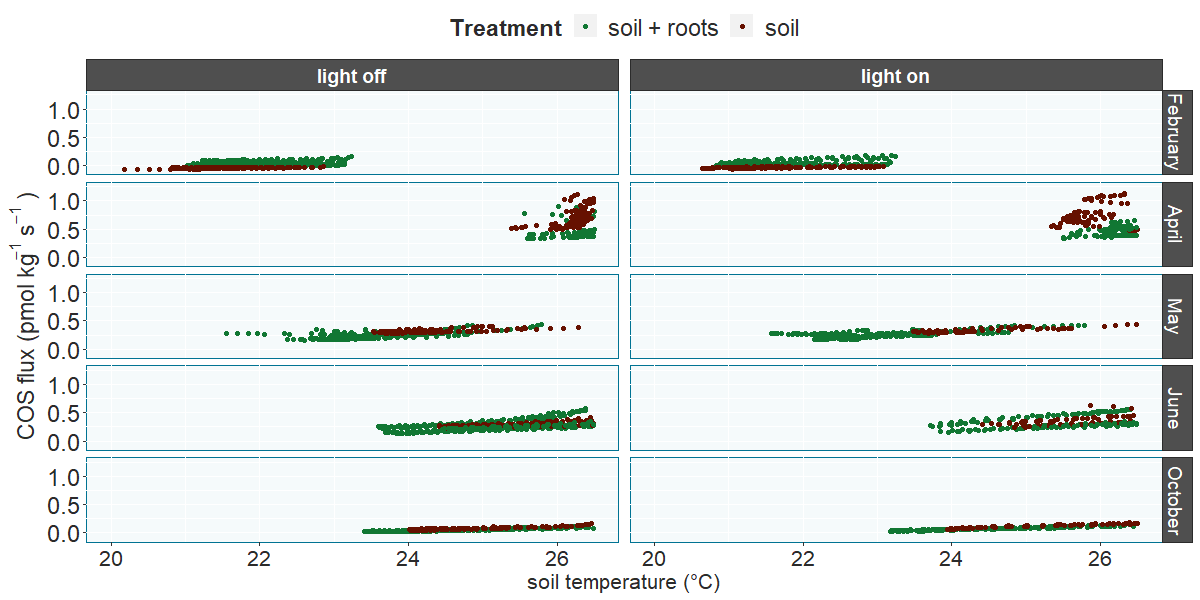


**Suppl. Figure 4** COS fluxes vs soil temperature for each month separated in growth lamp on and off.
